# Supplementary material for: Correlation between Serum 25-Hydroxyvitamin D Level and Peripheral Arterial Stiffness in Chronic Kidney Disease Stage 3–5 Patients
Source: Nutrients. 2022 Jun 11;14(12):2429. doi: 10.3390/nu14122429 (PMC9227485; doi:10.3390/nu14122429)
Supplement: Supplementary file 1 [file nutrients-14-02429-s001.zip › nutrients-1746787-supplementary.pdf]

Supplementary materials

Table S1. Correlation and linear regression of left brachial-ankle pulse wave velocity and clinical variables among 180 patients with chronic kidney disease stage 3–5.

Variables	Left brachial-ankle pulse wave (m/s)				
	Simple Linear Regression		Multivariable Linear Regression		
	r	*p* Value	Beta	Adjusted R^2 change	*p* Value
**Female**	0.036	0.627	—	—	—
Diabetes mellitus	0.117	0.118	—	—	—
Hypertension	0.083	0.267	—	—	—
Age (years)	0.449	< 0.001*	0.542	0.197	< 0.001*
Body mass index (kg/m^2)	0.060	0.424	—	—	—
Systolic blood pressure (mmHg)	0.379	< 0.001*	—	—	—
Diastolic blood pressure (mmHg)	0.287	< 0.001*	0.370	0.172	< 0.001*
Total cholesterol (mg/dL)	0.028	0.708	—	—	—
Log-triglyceride (mg/dL)	−0.050	0.507	—	—	—
LDL-C (mg/dL)	0.016	0.934	—	—	—
Log-glucose (mg/dL)	0.059	0.432	—	—	—
Log-BUN (mg/dL)	−0.033	0.656	—	—	—
Log-creatinine (mg/dL)	0.043	0.566	—	—	—
eGFR (mL/min)	−0.143	0.056	—	—	—
Total calcium (mg/dL)	−0.005	0.944	—	—	—
Phosphorus (mg/dL)	0.043	0.569	—	—	—
Log-iPTH (pg/mL)	0.131	0.079	—	—	—
Log-CRP (mg/dL)	0.195	0.009*	—	—	—
25-hydroxyvitamin D (ng/mL)	−0.376	< 0.001*	−0.297	0.082	< 0.001*

Data of triglyceride, glucose, BUN, creatinine, iPTH, and C-reactive protein levels showed skewed distribution, and therefore were log-transformed before analysis. Analysis of data was performed using the Pearson or Spearman correlation and multivariable stepwise linear regression analysis (adapted factors were age, systolic blood pressure, diastolic blood pressure, log-CRP and 25-hydroxyvitamin D). HDL-C, high-density lipoprotein cholesterol; LDL-C, low-density lipoprotein cholesterol; BUN, blood urea nitrogen; eGFR, estimated glomerular filtration rate; iPTH, intact parathyroid hormone; CRP, C-reactive protein. *$p < 0.05$ was considered statistically significant.

Table S2. Correlation and linear regression of right brachial-ankle pulse wave velocity and clinical variables among 180 patients with chronic kidney disease stage 3–5.

Variables	Right brachial-ankle pulse wave (m/s)				
	Simple Linear Regression		Multivariable Linear Regression		
	r	*p* Value	Beta	Adjusted R^2 change	*p* Value
Female	0.039	0.604	—	—	—
Diabetes mellitus	0.116	0.121	—	—	—
Hypertension	0.103	0.169	—	—	—
Age (years)	0.459	< 0.001*	0.550	0.206	< 0.001*
Body mass index (kg/m^2)	0.046	0.538	—	—	—
Systolic blood pressure (mmHg)	0.342	< 0.001*	—	—	—
Diastolic blood pressure (mmHg)	0.268	< 0.001*	0.363	0.158	< 0.001*
Total cholesterol (mg/dL)	0.068	0.367	—	—	—
Log-triglyceride (mg/dL)	-0.055	0.463	—	—	—
LDL-C (mg/dL)	0.068	0.361	—	—	—
Log-glucose (mg/dL)	0.080	0.287	—	—	—
Log-BUN (mg/dL)	-0.018	0.812	—	—	—
Log-creatinine (mg/dL)	0.049	0.512	—	—	—
eGFR (mL/min)	-0.151	0.043*	—	—	—
Total calcium (mg/dL)	0.001	0.999	—	—	—
Phosphorus (mg/dL)	0.051	0.500	—	—	—
Log-iPTH (pg/mL)	0.110	0.140	—	—	—
Log-CRP (mg/dL)	0.175	0.019*	—	—	—
25-hydroxyvitamin D (ng/mL)	−0.322	< 0.001*	−0.244	0.055	< 0.001*

Data of triglyceride, glucose, BUN, creatinine, iPTH, and C-reactive protein levels showed skewed distribution, and therefore were log-transformed before analysis. Analysis of data was performed using the Pearson or Spearman correlation and multivariable stepwise linear regression analysis (adapted factors were age, systolic blood pressure, diastolic blood pressure, eGFR, log-CRP and 25-hydroxyvitamin D). HDL-C, high-density lipoprotein cholesterol; LDL-C, low-density lipoprotein cholesterol; BUN, blood urea nitrogen; eGFR, estimated glomerular filtration rate; iPTH, intact parathyroid hormone; CRP, C-reactive protein. *$p < 0.05$ was considered statistically significant.
